# The Effect of Aging on Physical Performance Among Elderly Manual Workers: Protocol of a Cross-Sectional Study

**DOI:** 10.2196/resprot.8196

**Published:** 2017-11-22

**Authors:** Kristoffer Larsen Norheim, Jakob Hjort Bønløkke, Afshin Samani, Øyvind Omland, Pascal Madeleine

**Affiliations:** ^1^ Physical Activity and Human Performance, SMI Department of Health Science and Technology Aalborg University Aalborg Denmark; ^2^ Department of Occupational Medicine Aalborg University Hospital Aalborg Denmark

**Keywords:** older workers, functional capacity, spirometry, inflammation, body composition, balance, force tracking

## Abstract

**Background:**

In 2012, the Danish Parliament decided to increase retirement age. Unfortunately, elderly people working in a physically demanding environment may be rendered unable to retain the ability to adequately perform the physical requirements of their jobs, due to age-related decreases in physical performance. Therefore, increasing the retirement age may not necessarily lead to the goal of keeping everybody in the labor market for a longer time. To date, our knowledge about the variations in physical performance of the elderly workforce is limited.

**Objective:**

In this cross-sectional study we seek to investigate the effects of aging on physical performance among elderly manual workers.

**Methods:**

Approximately 100 Danish manual workers between 50 and 70 years of age will be recruited. The main measurement outcomes include: (1) inflammatory status from blood samples; (2) body composition; (3) lung function; (4) static and dynamic balance; (5) reaction time, precision, and movement variability during a hammering task; (6) handgrip strength, rate of force development, and force tracking; (7) estimated maximal rate of oxygen consumption; and (8) back mobility. Additionally, information regarding working conditions, physical activity levels, and health status will be assessed with a questionnaire.

**Results:**

Data collection is expected to take place between autumn 2017 and spring 2018.

**Conclusions:**

This study will increase the knowledge regarding variations in physical performance in the elderly workforce and may identify potential workplace hazards. Moreover, this study might shed light on the potentially problematic decision to increase retirement age for all Danish citizens.

## Introduction

It is expected that the world’s proportion of elderly people above the age of 60 years will almost double within the next 35 years [1]. At this time, approximately one fourth of the Danish population is above the age of 65 years [2]. Due to a longer life expectancy than seen in the past, in 2012 the Danish Parliament decided to increase retirement age [3], thereby increasing the number of elderly workers. In practice, this means that individuals born after 1966 can expect to be at least 69 years old at the time of retirement. The increased life expectancy was one of the central arguments; however, this may be problematic as increased lifespan is caused by other factors than delayed onset of the aging processes, such as healthier nutrition, better living standards and education, and public-health efforts [4]. Hence, elderly people working in a physically demanding environment (eg, building and constructions sites) may be rendered unable to retain the ability to adequately perform the physical requirements of their jobs, due to age-related decreases in physical performance. In healthy elderly people, increased longevity may lead to a postponed retirement age, but that is far from certain for people with chronic conditions such as osteoarthritis and osteoporosis. Therefore, increasing the retirement age may not necessarily lead to the goal of keeping everybody in the labor market for a longer time [5]. Knowledge about the variations in physical performance of the elderly workforce when physically challenged is limited [6-8]. Accordingly, the effects of an increased retirement age among elderly manual workers are difficult to predict.

Aging is associated with a gradual loss of muscle mass, strength, and power [9-11], in addition to reductions in maximal rate of oxygen consumption (VO_2max_) [12], lung capacity [13,14], and impairment of postural control [15]. Reductions in VO_2max_ have been observed to be approximately 10% per decade after the age of 30 (a process which seems to accelerate further after the age of 70 [16-18]), whereas forced expiratory volume in 1 second (FEV_1_) declines approximately 20 mL annually from the age of 25 years [19]. This annual decline increases to 38 mL after the age of 65 years [14], which means that on average a person at the age of 70 has lost 1 L in FEV_1_ over the preceding 45 years. Concomitant to these changes, forced vital capacity (FVC) is reduced at a slightly slower speed [19]. Furthermore, the age-related reductions in muscle strength affects both inspiratory and expiratory muscles [20], possibly inhibiting ventilation during physical activity.

Contrary to the age-related loss of respiratory capacity, skeletal muscle strength is relatively well preserved until the age of 50 years. From this age, the annual decline in strength is approximately 1-2%, whereas muscle power is lost at an even faster rate [11,21]. Regarding rate of force development (RFD), a previous study demonstrated that a significant loss of rapid force capacity would be evident after the fifth decade, while loss of maximal strength would be more pronounced after the sixth decade of life [22]. Some authors argue that RFD is of more functional significance in elderly people compared to maximal strength because rapid force production is more related to tasks such as postural control and grabbing a rail to regain balance and prevent falls [23]. Conversely, daily activities that involve the manipulation of objects rarely require maximal force or explosive force, but rely mostly on submaximal force production. Hence, measurements of submaximal force production in both static and dynamic conditions (eg, force tracking) may provide additional information about force steadiness and coordination [24], which is needed during activities of daily living at work and during leisure.

Sarcopenia, also known as the age-related loss of muscle mass, typically starts from the end of the fifth decade of life [9,25], and may sometimes be accompanied by an increase in fat mass [26]. Thus, body mass may remain somewhat stable in the elderly [27]; however, a negative change in body composition may have detrimental consequences [28]. Given that people with physically demanding occupations are required to be physically active throughout a workday, a decrease in muscle mass together with an increase in fat mass would be especially problematic in this population. Another challenge for elderly individuals is that comorbidity (ie, having more than one chronic condition) increases with age [29]. Additionally, some conditions may interact, thereby exacerbating the negative impact of a disease on quality of life and work ability. For instance, chronic obstructive pulmonary disease is usually accompanied by skeletal muscle weakness, musculoskeletal disorders, osteoporosis, and chronic inflammation [30,31]. Moreover, the prevalence of hip and knee osteoarthritis increases with age, while physically demanding work may accelerate the development of this pathology [32]. Several risk factors have been proposed to explain this interaction, including age and physical inactivity. The exact pathogenesis contributing to these comorbidities, together with the treatment strategies, are not clearly established. Interestingly, systemic inflammation, which is typically elevated in patients with chronic obstructive pulmonary disease, is not only associated with an age-related decline in lung function [33], but is also negatively associated with muscle mass in elderly people [34]. These findings call for research initiatives assessing both the respiratory and the musculoskeletal system in relation to occupational exposures, and other risk factors such as body composition and inflammatory status.

Balance is typically compromised in elderly people. Although postural control is largely related to muscle strength and the ability to produce rapid force, it also requires an integration of the information from the visual, vestibular, and somatosensory systems to generate the appropriate motor response to maintain static and dynamic balance [15,35]. Thus, a combination of the alterations in the musculoskeletal system and the afferent information from sensory systems are responsible for the reduced ability to maintain motor coordination (including balance) among the elderly [35]. Paradoxically, although visual acuity is progressively compromised with increasing age, the reliance on the visual system to maintain postural control increases with age, especially when balance is challenged [35]. These changes may therefore result in impairments in physical performance and, as a result, the ability to sustain the required level of efficiency in a physically demanding job. Hence, the physiological changes in relation to aging may challenge the ability of elderly manual workers to maintain an adequate level of performance in their workplace. Moreover, these changes may result in elderly manual workers working closer to their maximal capacity on a daily basis, which may result in an increased risk of developing musculoskeletal disorders [36,37].

To date, we know too little about how physically demanding work affects physical performance and how declining physical performance affects the ability to perform physically demanding work in elderly populations. Specifically, more information is needed about how different work exposures, health statuses, and physical activity levels relate to different measures of physical performance. Although some of the reductions in physical performance might begin as early as the third decade of life, the most pronounced impairments occur starting from the fifth decade. Thus, information regarding the variations in physical performance among elderly manual workers in the last two decades of working life is of importance, so that recommendations related to their work environment can be made. Lastly, chronological age (ie, time since birth) may well be an important risk factor for several adverse outcomes; however, all people age differently. The term *biological age* has been used to describe a person’s health status, thereby giving a better prediction of physical capacity later in life than chronological age [38]. Although a single marker of biological age has not been discovered, a combination of several putative biomarkers (including handgrip strength, standing balance, muscle mass, inflammatory status, and lung function) have been suggested to create a model for determining biological age [39,40]. Hence, the present study will enable us to not only investigate the effect of chronological age on physical performance, but may also give insight into the biological age of elderly manual workers.

The present paper describes the study protocol for a study that aims to investigate variations in physical performance among elderly Danish manual workers aged 50 to 70 years. Our primary outcomes include handgrip strength, FEV_1_, and FVC. Secondary outcomes include force steadiness, reaction time, aerobic capacity, motor coordination, balance, body composition, back mobility, and inflammatory status. Tertiary outcomes are self-reported levels of physical activity, health status, and different work exposures. This is the first study to combine measurements of respiratory and musculoskeletal systems in elderly manual workers, and the collected data will therefore increase our knowledge regarding the elderly workforce. The main research questions in the study are:

1. To what extent does physical performance change in elderly manual workers with physically demanding occupations during the last two decades of working life?
2. To what extents do work environment and physical activity levels predict respiratory and musculoskeletal function in elderly manual workers?

Based on the available literature, we hypothesize that physical performance is negatively associated with age, and that physical work magnifies this negative relationship when compared to the general population. Thus, we expect work environment and physical activity to be predictive of respiratory and musculoskeletal function in this cohort.

## Methods

### Study Design

**Figure 1:**
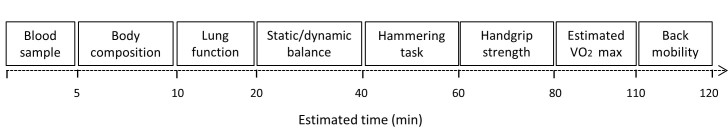
Test order and estimated time.

The present study is a cross-sectional investigation which explores the variations in physical performance in elderly (aged 50-70 years) Danish manual workers working in a physically demanding trade. Data collection is expected to take place between autumn 2017 and spring 2018. All tests will be conducted in a research laboratory at Aalborg University, Denmark. Each experimental session is expected to last for approximately 2 hours. The order in which the tests will be conducted is the following: (1) inflammatory status will be measured from venous blood samples; (2) anthropometrics (ie, height, weight) and blood pressure will be measured, followed by an estimation of body composition based on bioelectrical impedance analysis (BIA); (3) lung function will be measured using spirometry; (4) static and dynamic balance will be measured on a force platform during quiet standing and a sit-to-stand motion; (5) reaction time, precision, and movement variability will be measured during a hammering task; (6) handgrip strength, RFD, and force tracking will be measured with a hand dynamometer; (7) estimated VO_2max_ will be measured on a bicycle ergometer; and (8) back mobility (Figure 1).

### Recruitment of Participants

We aim to include approximately 100 manual workers aged 50-70 years. The study subjects will be recruited from a questionnaire sent out to more than 5000 Danish manual workers as a part of the ALdring og Fysisk Arbejde cohort (ALFA; Aging and Physical Work), which was created from a register-based cohort of all manual workers in Denmark aged 39 years and older in 1999 (n=155,358). Briefly, the questionnaire included 86 items, mostly regarding work and working conditions, but also 23 items regarding health. Lastly, the questionnaire included a question asking if the subject would like to participate in a clinical study of physical performance. Those who respond in the affirmative to this question will be contacted via email. The selection will aim to ensure representability over the age range by recruiting in bins of 5 years from 50 to 70 years. Subjects with musculoskeletal disorders, osteoarthritis, cardiovascular disease, or any other health condition that contradicts physical testing will be excluded from tests they cannot safely perform. Hence, hypertensive subjects will not complete the cycling test, whereas subjects with severe shoulder pain will not complete the hammering test. All participants will be informed about the purpose of the study and will give written informed consent to participate in the clinical examination. The study will be carried out in accordance to the Helsinki declaration and is approved by the ethics committee of region North Jutland (N-20160023).

### Measurements

#### Biochemistry

Blood will be drawn from the antecubital vein into 6 mL ethylenediamine tetraacetic acid tubes followed by centrifugation and extraction of plasma. Plasma will be used for high sensitivity analysis of C-reactive protein with a latex particle-enhanced immunoturbidimetric assay, and interleukin-6 will be analyzed by an enzyme linked immunosorbent assay.

#### Bioelectrical Impedance Analysis

Body composition will be estimated using a direct segmental multi-frequency bioelectrical impedance machine (InBody 370, Biospace). The apparatus uses three frequencies (5, 50, and 250 kHz) at five body segments (right arm, left arm, trunk, right leg, and left leg), with a test duration of approximately 45 seconds. The main measurement outcomes include fat-free mass, fat mass, and percent body fat. Although not recognized as the *gold standard* for estimation of body composition, BIA is widely used in large cohort-based and cross-sectional studies [41] due to its low cost and noninvasive technique. Several studies have found direct segmental multi-frequency BIA to be a valid tool for assessment of body composition in both young and older men and women [42,43].

#### Spirometry

Basal lung function will be measured using a Spirobank II SMART (Medical International Research [MIR], Rome, Italy) spirometer, disposable MIR turbine flowmeters, and MIR winspiroPRO software (version 6.5.0). The main outcomes include FEV_1_, FVC, and peak expiratory flow, which will be conducted as recommended by The European Respiratory Society [44] and The American Thoracic Society [45] standards. Briefly, from a standing position the subjects will be asked to inhale fully, place the spirometer in their mouth, immediately (<1 second) followed by a forced maximal expiration, which is ended when they are unable to expire more air, or after at least 6 seconds. A nose clip will be worn during the testing. The subjects will be asked to avoid pursing their lips, closing their teeth around the spirometer, or letting any air leak between their lips and the mouthpiece during the expiration. Each subject will perform a minimum of three trials and a maximum of eight trials until at least three satisfactory trials have been performed, and the difference between the highest and the second highest FVC or FEV_1_ is no more than 150 mL [44]. The spirometer will be calibrated daily using a 3 L calibrated airtight syringe.

#### Static and Dynamic Balance

Static and dynamic balance will be measured during quiet standing and during a sit-to-stand motion on a force platform (AMTI AccuSway, Watertown, MA, USA). Static balance will be assessed during three conditions: first during quiet standing with eyes open; second during quiet standing with eyes closed; and third during quiet standing with eyes open, while counting backwards from 30 in multiples of three (ie, 30, 27, 24, and so on) to increase cognitive load. In each of the above-mentioned conditions the subjects will be asked to stand for 1 minute, which will be repeated three times for each condition, and they will be instructed to stand as quietly as possible [46]. Dynamic balance will be assessed with the subjects completing a sit-to-stand motion from a chair with five rises as fast as possible. This sit-to-stand test is a part of the short physical performance battery and is used to assess lower extremity function [47]. Spatial-temporal changes in displacement of the center of pressure (CoP) will be computed in the anterior-posterior and medial-lateral directions. Additionally, the standard deviation and sample entropy of the CoP will be computed, as described previously [48].

#### Hammering Task

**Figure 2:**
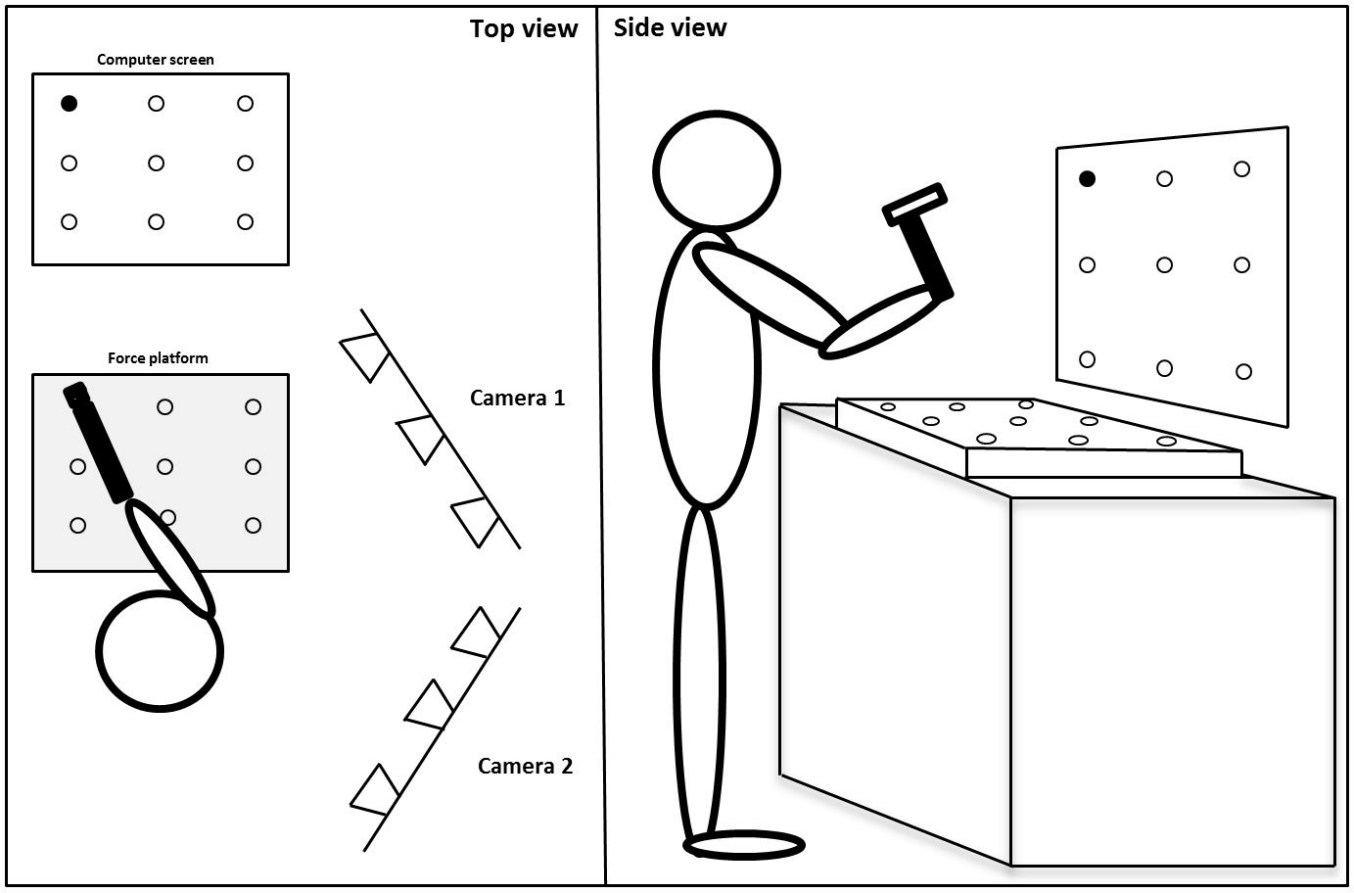
Illustration of experimental set-up during the hammering task.

Reaction time, precision, and movement variability will be measured using a force platform (AMTI AccuSway, Watertown, MA, USA), a computer screen for visualization, and a rubber hammer (Figure 2). From a standing position, subjects will be instructed to hammer as fast and precisely as possible on the correct mark on the force platform, as visualized on the computer screen. The program will be set to randomly (and with equal probability) indicate one out of nine marks at 1-second intervals. Active markers will be attached to the hammer and the dominant arm. The hammering motion will tracked by the Visualeyez II system set up with two VZ4000 trackers (Phoenix Technologies Inc., BC, Canada) and sampled at 100 Hz. The movement will then be decomposed in three dimensions using Euler angles. All recordings will be synchronized by the end of frame pulse generated by the motion capture system indicating the first frame of the recordings. Movement amplitude and variability will be computed, as described previously [49].

#### Handgrip Strength

Maximal handgrip strength, RFD, and force tracking will be measured using a digital hand dynamometer (Model G100, Biometrics Ltd, Gwent, UK). The subjects will be seated in a chair with their lower arms resting on an armchair (90-degree angle in the elbow) while holding the dynamometer. The test begins with the subjects generating their maximal force onto the dynamometer and ends when the force starts to decline. Three trials will be performed using the dominant hand. Thereafter, the subjects will perform an endurance trial using their dominant hand, in which they are asked to exert 20% of their maximal force until task failure, which is defined as an inability to maintain the output force within 2% of maximal force around the set value. Maximal grip strength (measured as peak force in Newtons) and RFD (measured as the rate of force rise [change in peak force/change in time] in Newtons per second) will be calculated during the maximal contractions, whereas the standard deviation (absolute variability), coefficient of variation (relative variability), and the sample entropy (structural variability) of the force signal will be computed during the submaximal trial, as described previously [50]. Measurement of handgrip strength has previously been shown to be a reliable measure of strength in the upper extremities [51] and low levels of handgrip strength are reported to be a strong predictor of disability and mortality, and a marker of sarcopenia [52,53].

#### Estimated Maximal Rate of Oxygen Consumption

The estimated VO_2max_ test will be conducted using a bicycle ergometer (Monark AB, Varberg, Sweden) and a Polar A300 heart rate monitor (Polar Electro Oy). Instructions will be given, as recommended by the Danish Health Authority [54]. Briefly, the cycle ergometer will be adjusted to the individual subject, followed by an 8-minute warm-up on a low resistance (measured in Watts) that increases the subject’s heart rate approximately 20 beats/minute from resting levels. The resistance will then be increased to a level that raises the heart rate of the subject to somewhere between 130-160 beats/minute. Heart rate will be registered every 30 seconds and the test will be terminated when the heart rate has been stable for at least 2 minutes. The criterion for a stable heart rate is less than 4 beats/minute change, as measured every 30 seconds for 2 minutes, and the test will continue until this criterion is met. The average heart rate across the four last measures, together with the selected ergometer resistance, will be used to estimate VO_2max_ using the Åstrand nomogram and linear extrapolation, with correction for gender [55]. This method has shown a high degree of precision [56].

#### Back Mobility

Back mobility will be measured using the fingertip-to-floor (FTF) test. The FTF test is a reliable measure used to assess forward mobility of the spine and pelvis [57], and it is able to predict changes in disability in patients with lower back pain [58]. The subjects will be standing barefoot on an elevated platform. Keeping their knees fully extended, subjects will bend forward and reach as low as possible with their arms. The vertical distance between the platform and the tip of their middle finger will be noted. This value can be both positive and negative (ie, if their fingers reach below the platform the value will be noted as negative in centimeters) [57].

#### General Health and Work Ability

The answers to the ALFA questionnaire, which includes questions about general health, work ability, and environment, as well as social and psychological wellbeing, will be used as covariates in this study. To get a current assessment of some of these covariates, a shorter version of the questionnaire will be answered at the time of the clinical examinations. This short questionnaire will include questions about general health, leisure-time physical activity, pain, and work ability (see Multimedia Appendix 1).

### Statistics

All continuous data will be tested for normality using the Shapiro-Wilk test. Appropriate data transformation will be applied if normality is not met. Subject characteristics and descriptive results will be presented as means and standard deviations or standard errors, and percent distribution. Associations between age (dependent variable) and the measured outcomes (independent variables) will be analyzed with univariate linear regression models, whereas multivariate linear regression models will be constructed using backwards elimination (with adjustment for gender in both cases). When available, the measured outcomes will also be compared with normative data assessing the general population in this age group. Pearson’s Chi-square tests will be used to assess the probability of independency between different self-reported work exposures (eg, work experience, seniority, heavy lifts in the workplace) and the measured outcomes (made categorical by dividing outcomes into *high* or *low*, based on either available reference values [59] or cluster analysis). In models with categorical variables, odds ratios will be calculated and statistical significance levels will be set *a priori* to *P*<.05.

### Sample Size

Our primary outcomes are handgrip strength, FEV_1_, and FVC. Based on previous studies, we expect age to be a stronger predictor of handgrip strength compared to FEV_1_ or FVC; hence, the latter was used to calculate sample size. Considering three predicators (age, height, smoking) describing the primary outcome (FEV_1_ or FVC) variations with a medium average correlation between each of the predictors and the outcome (rho=0.3), and a low average correlation between the predictors (rho=0.1), the sample size was calculated to be 102 subjects in a multiple regression analysis to obtain 5% type I and 20% type II errors [60].

## Results

Subjects are currently being contracted via telephone and email. It is expected that data collection will be completed by March 2018.

## Discussion

In several jobs, the physical demands for elderly workers are at the same level as for younger workers [37,61,62]. Due to a potential decrease in working capacity, the resulting workload may change from an acceptable load into daily physical *overload*, which might result in negative long-term health effects, such as chronic musculoskeletal symptoms [63,64], work absenteeism, and early pension retirement. Physical overload is often related to the positive adaptive responses of endurance and resistance training. Most studies, however, find no training effect of prolonged exposure to heavy manual labor [65-68]. Therefore, a more detailed understanding of the effects of age on physical performance in elderly manual workers will provide additional knowledge regarding issues related to changes in physical performance, confounding factors (eg, smoking, work environment, health status), and the balance between physical workloads and physical work capacity. Some studies have investigated the effect of age on physical performance among elderly workers; however, these studies have either been restricted by testing relatively young (<60 years) workers [65,67,69,70] or by assessing only a limited number of parameters [68,71]. Therefore, an extensive test-battery with direct measures of respiratory capacity and musculoskeletal function in the oldest of workers, along with health profiles and work exposures, will enable us to identify associations between age and physical performance in this specific population. Moreover, this study might shed light on the potentially problematic decision to increase retirement age.

### Strengths and Limitations

The present study is strengthened by the objective and extensive clinical examination of physical performance employed in a relatively understudied group. One of the limitations of this study includes the use of a cross-sectional design. Specifically, cross-sectional studies investigating alterations in physical performance with increasing age tend to find different changes compared to findings in longitudinal studies. Such discrepancies may stem from several issues, including differences in working conditions, environmental factors, and research methodologies [37]. However, the financial and time-saving advantages of cross-sectional studies may be compensatory and should still give insight into areas for future longitudinal investigations. Moreover, establishing this cohort will enable us to conduct follow-up studies in the future. Another limitation may be that the oldest workers recruited for this study represent a highly selected cohort. The “average” elderly manual worker may have already (prematurely) withdrawn from the workforce, thereby leaving only the older workers with the highest physical performance in the labor market. This issue is known as the *healthy worker effect* [72,73], and should be kept in mind when interpreting our findings.

## Appendix

Multimedia Appendix 1 Questionnaire.

## Abbreviations

ALFA: ALdring og Fysisk Arbejde
BIA: bioelectrical impedance analysis
CoP: center of pressure
FEV1: forced expiratory volume in 1 second
FTF: fingertip-to-floor
FVC: forced vital capacity
MIR: Medical International Research
RFD: rate of force development
VO_2max_: maximal rate of oxygen consumption
