# Supplementary material for: The Effect of Aging on Physical Performance Among Elderly Manual Workers: Protocol of a Cross-Sectional Study
Source: JMIR Res Protoc. 2017 Nov 22;6(11):e226. doi: 10.2196/resprot.8196 (PMC5719227; doi:10.2196/resprot.8196)
Supplement: Multimedia Appendix 1 [file resprot_v6i11e226_app1.pdf]

Do you smoke?

(1) ☐ Yes
(2) ☐ Have smoked, but not anymore and stopped more than 2 years ago
(3) ☐ Have smoked, but not anymore and stopped less than 2 years ago
(4) ☐ Have never smoked

How many years have you smoked? ___________

How much do you/did you smoke per day on average?

Cigarettes / cheroots / pipe per day: _______________

Are you currently working?

(1) ☐ Yes
(2) ☐ No

How many hours do you work per week? _____________________

When were you finished training? _________________________________

What is your craft? ____

How many days have you had difficulty (pain or discomfort) within the last 12 months in each of the following body regions?

	**0 days**	**1-7 days**	**8-30 days**	**31-90 days**	**>90 days**	**Every day**
Neck/shoulder	(1) ☐	(2) ☐	(3) ☐	(4) ☐	(5) ☐	(6) ☐
Arm (elbow, wrist and hand)	(1) ☐	(2) ☐	(3) ☐	(4) ☐	(5) ☐	(6) ☐
Lower back	(1) ☐	(2) ☐	(3) ☐	(4) ☐	(5) ☐	(6) ☐
Hip	(1) ☐	(2) ☐	(3) ☐	(4) ☐	(5) ☐	(6) ☐
Knee	(1) ☐	(2) ☐	(3) ☐	(4) ☐	(5) ☐	(6) ☐

Indicate the degree of difficulty (pain or discomfort) within the last 7 days in each of the following body regions.

	**0**	**1**	**2**	**3**	**4**	**5**	**6**	**7**	**8**	**9**	**10**
Neck/shoulder	(1) ☐	(2) ☐	(3) ☐	(4) ☐	(5) ☐	(6) ☐	(7) ☐	(8) ☐	(9) ☐	(10) ☐	(11) ☐
Arm (elbow, wrist and hand)	(1) ☐	(2) ☐	(3) ☐	(4) ☐	(5) ☐	(6) ☐	(7) ☐	(8) ☐	(9) ☐	(10) ☐	(11) ☐
Lower back	(1) ☐	(2) ☐	(3) ☐	(4) ☐	(5) ☐	(6) ☐	(7) ☐	(8) ☐	(9) ☐	(10) ☐	(11) ☐
Hip	(1)	(2)	(3)	(4)	(5)	(6)	(7)	(8)	(9)	(10)	(11)

	0	1	2	3	4	5	6	7	8	9	10
	☐	☐	☐	☐	☐	☐	☐	☐	☐	☐	☐
Knee	(1) ☐	(2) ☐	(3) ☐	(4) ☐	(5) ☐	(6) ☐	(7) ☐	(8) ☐	(9) ☐	(10) ☐	(11) ☐

Respiratory disorders

Has a doctor told you that you are suffering from chronic obstructive pulmonary disease (COPD)?

(1) ☐ Yes
(2) ☐ No

Has a doctor told you that you are suffering from asthma?

(1) ☐ Yes
(2) ☐ No

Has a doctor told you that you are suffering from other respiratory disorders?

(1) ☐ Yes
(2) ☐ No

If yes, what is the name of the disorder?

Musculoskeletal symptoms

Do you have any injuries in the following parts of your body?

	Yes	No
Neck/shoulder	(1) ☐	(2) ☐
Back	(1) ☐	(2) ☐
Arms	(1) ☐	(2) ☐
Legs	(1) ☐	(2) ☐

Do you suffer from the following diseases or any aftereffects of disease?

	Yes	No
High blood pressure	(1) ☐	(2) ☐
Chest pain during physical exercise	(1) ☐	(2) ☐
Diabetes	(1) ☐	(2) ☐
Aftereffects of blood clots in the heart	(1) ☐	(2) ☐

Leisure-time physical activity

How many hours per week have you spent on average on each of the following leisure-time activities in the last year?
Include transport to and from work

	More than 4 hours/week	2-4 hours/week	Less than 2 hours/week	I don´t do this activity
Walking, biking or other low-intensity exercise, where you do not get short of breath nor sweat (e.g. Sunday walks or low-intensity gardening)	(1) ☐	(2) ☐	(3) ☐	(4) ☐
Exercise training, heavy gardening or higher-intensity walking/biking, where you do sweat and get short of breath	(1) ☐	(2) ☐	(3) ☐	(4) ☐
Strenuous exercise training or competitive sports	(1) ☐	(2) ☐	(3) ☐	(4) ☐

Work ability

How do you rate your current work ability with respect to the physical demands of your work?

(1) ☐ Poor
(2) ☐ Fair
(3) ☐ Good
(4) ☐ Very good
(5) ☐ Excellent
